# Recombinant B2L and Kisspeptin-54 DNA Vaccine Induces Immunity Against Orf Virus and Inhibits Spermatogenesis In Rats

**DOI:** 10.1038/s41598-019-52744-y

**Published:** 2019-11-07

**Authors:** Teketay Wassie, Zeng Fanmei, Xunping Jiang, Guiqiong Liu, Shishay Girmay, Zhang Min, Liu Chenhui, Dong Dong Bo, Sohail Ahmed

**Affiliations:** 1 Key Laboratory of Agricultural Animal Genetics, Breeding and Reproduction of Ministry of Education, Huazhong Agricultural University, Wuhan, 430070 People’s Republic of China; 2 Laboratory of Sheep and Goat Genetics, Breeding and Reproduction, College of Animal Science and Technology, Huazhong Agricultural University, Wuhan, People’s Republic of China

**Keywords:** DNA vaccines, DNA vaccines

## Abstract

Orf is a highly contagious zoonotic disease of small ruminants caused by Parapoxvirus. Kisspeptin, encoded by the KISS1 gene with its cognate receptor GPR-54 is recognized as an upstream orchestrator in the hypothalamic-pituitary-gonadal axis. This study was designed to construct a DNA vaccine that produces a fused peptide composed of a major immunodominant protein of the orf virus (B2L) and kisspeptin-54, a neuropeptide with recognized roles in mammalian reproductive biology. The administration of this recombinant vaccine is shown to produce a significant antibody and cell-mediated immune response directed against B2L compared to the control group (*p* < 0.05). Furthermore, we found that rats inoculated with PBK-asd vaccine up-regulated antigen-mediated splenocyte proliferation and significantly raised antigen-specific tumor necrosis factor-alpha (TNFα-), interferon-gamma (IFN-ϒ) and interleukin (IL-2) compared to the control group (*p* < 0.05). This recombinant vaccine also stimulated antibody responses to kisspeptin and decreased serum luteinizing hormone and testosterone levels. Moreover, the current recombinant vaccine caused testicular atrophy and arrested spermatogenesis. It is concluded that this recombinant B2L and Kisspeptin-54 vaccine could be a promising approach for construction of bivalent orf virus and immunocastration vaccine. Furthermore, we concluded that the orf virus envelope protein (B2L) could be used as an immunomodulator for kisspeptin-54 to produce a strong antibody response.

## Introduction

Orf is a highly contagious disease of small ruminants caused by Parapoxvirus. It is a zoonotic disease in which human contract the virus via contact with diseased animals^1^. Orf virus infection results in erythema, vesicles or pustules and formation of scabs in the infected animals^2^. Lloyd *et al*.^3^ reported that the humoral antibody produced by infected sheep plays role to reduce viral replication in their skin.

Immunization is the cheapest way to control and prevent most infectious diseases, especially those that cannot be treated. Vaccination of the host animals is the ultimate choice for controlling the orf virus because there is no effective antiviral treatment for this disease^4^. Despite currently there is a licensed live attenuated orf virus vaccine, it is unable to induce long-lasting immunity and unable to protect the host from reinfection^5^, due to the presence of the immunomodulator genes that inactivate the host immune response^6^. Therefore, the rational vaccination strategies of orf virus should be devised to target the virus immunomodulators gene to induce robust and long-lasting immunities^7^. In this regard, there has been one attempt to construct a DNA vaccine using highly envelope proteins of orf virus, showed a promised approach to developing a DNA vaccine against orf disease in experimental mice^8^.

The orf virus encodes 134 genes among which the orf virus B2L gene is a major and highly immunogenic envelope protein^9^. Data from the mouse model demonstrated that the efficacy of the influenza vaccine could be improved by co-administering the vaccine with the B2L gene^10^, suggesting that the B2L gene can be used in combination with other genes. Furthermore, Yogisharadhya *et al*.^11^ reported that B2L protein could activate the animal’s immune system and is therefore useful as an immunomodulator in veterinary vaccine preparations. Thus, constructing a DNA vaccine against orf disease using the B2L gene could be reasonable. Studies on lambs demonstrated that virus-specific antibodies and cell-mediated immune responses, particularly CD4^+^ T lymphocytes are essential for reducing orf virus replication in the infected skin^3^, suggesting that vaccination strategies should target for the activation of these immune systems.

Reproduction, which is an essential feature for the survival of an organism, is coordinated by the complex neuroendocrine dynamic interplay of the hypothalamic-pituitary-gonadal (HPG) axis. Castration is performed in the livestock industry aimed at improving meat quality and also reducing aggressive behavior, and controlling undesirable breeding. Since long years ago, livestock producers have practiced different methods of castration like mechanical castration using elastrator band and burdizzo, and surgical castration. Recently, immunocastration targeting reproductive hormones in the HPG axis and developing a vaccine against them anticipated as an alternative to surgical castration^12^.

Until 2003, gonadotropin-releasing hormone (GnRH) was known to be the top upstream hormone on the HPG axis, which regulates reproduction through its potent effects on downstream follicle- stimulating hormone (FSH) and luteinizing hormone (LH) secretion. Based on this milieu, to date, compelling studies in different species of animals showed that immunocastration against GnRH hormone has been a promising approach to inhibit reproduction and could be used as an alternative to surgical castration^13–15^. However, in this complex HPG axis, the hypothalamic KISS1 and its ligand G protein-coupled receptor (GPR-54) system have recently been recognized as a critical modulator of GnRH secretion and pubertal onset^16^.

Previous experimental data showed that kisspeptin administration induced gonadotropin secretion in the rat^17^, sheep^18^ and human^19^. Recent pharmacological evidence also revealed that kisspeptin could potentially be used as a treatment for fertility disorders in humans^20,21^. Considering the pivotal role of kisspeptin as a major elicitor of GnRH/LH secretion acting upstream on GnRH, we hypothesized that kisspeptins might be a potential area for the endocrine intervention of the HPG axis. In this regard, previous breakthrough research has been conducted in our laboratory by fusing KISS1 with HBsAg-S gene, indicating that KISS1 is a potential target to develop immunocastration vaccine^22,23^.

Although numerous aspects of kisspeptin’s role as a critical regulator of reproduction are well known, data regarding the use of KISS1/kisspeptin system to synthesize vaccine against fertility is scant. Besides, the potential role of kisspeptin-54 as a recombinant bivalent vaccine development has not been previously identified.

Small molecules need an immunomodulator gene to be used as an antigen to activate the immune system to produce a strong antibody response. Hence, we used the orf virus B2L gene as an immunomodulator for kisspeptin-54. To our knowledge, recombinant orf virus B2L and kisspeptin-54 have not yet been constructed, and its efficacy as a bivalent vaccine has not been evaluated. Based on this background information, we designed a novel recombinant bivalent DNA vaccine by fusing major envelope protein of orf virus (B2L) with kisspeptin-54 that could induce immunity against B2L of orf virus and inhibit spermatogenesis.

## Materials and Methods

### Vaccine preparation

Competent cell x6097^asd−^ was stored in our laboratory. The PVAX-asd plasmid was prepared by excising the kanamycin antibiotic resistance gene from PVAX1 plasmid and replaced by aspartate-β- semialdehyde dehydrogenase (ASD) gene. The orf virus isolate MT-05 B2L gene, GenBank^TM^ accession number JN613809.1 was chemically synthesized (Wuhan Gene create Biological Engineering CO., LTD) and subcloned into PVAX-asd plasmid with *NheI* and *BamHI* restriction enzymes. The positive plasmid was identified by restriction digestion and confirmed by sequencing. Finally, the fusion plasmid labeled as PVAX-B2L-asd and stored in −20 °C. The recombinant B2L and kisspeptin-54 plasmid was constructed by subcloning the kisspeptin-54 into PVAX-B2L-asd plasmid. Briefly, the KISS1 gene (corresponding to aa 68–121; GenBank TM accession number NM_002256) and linker (G_4_S)_3_ was chemically synthesized (Wuhan Gene create Biological Engineering CO., LTD) and subcloned into PVAX-B2L-asd plasmid with *BamHI* and *EcoRI* restriction enzymes.

The (G_4_S)_3_ linker (*GGATCC*GGTGGAGGTGGCTCCGGTGGCGGAGGCTCTGGTGGAGGTGGCTCC*CTCGAG*) was used to improve and maintain the folding, expression, and biological activity of the fusion protein. Letters in italic at 5′ and 3′ end are *BamHI*, and *XhoI* restriction site, respectively. The plasmid was extracted using Tinaprep mini plasmid extraction kit (TIANGEN, Beijing, China). Finally, restriction endonuclease enzyme digestion and sequencing were used to confirm the insertion. This recombinant plasmid labeled as PVAX-B2L-(G_4_S) _3_-kisspeptin-54-asd (PBK-asd). The full map of the vaccine with their direction of insertion in the multiple cloning sites of PVAX1 vector is shown in Fig. S1. The PVAX-Kisspeptin-54 plasmid was constructed in the way that kisspeptin-54, synthesized by Wuhan Gene create Biological Engineering CO., LTD was subcloned into PVAX-asd plasmid at *XhoI* and *EcoRI* restriction sites. The positive plasmid was identified by restriction digestion, confirmed by sequencing and labeled as PK-asd.

### Detection of the protein expression

The Hela cells were cultured as previously described^22^. The cells were transfected with PBK-asd, PK-asd and PVAX-asd plasmids using Lipofectamine^TM^ 2000 Kit (Invitrogen, USA) in 6 well plates according to the manufacturer’s instruction. The cells were collected using RIPA after 48 hours of transfection and fractioned using 10% sodium dodecyl sulfate-polyacrylamide gel electrophoresis (SDS PAGE) and transferred to polyvinylidene fluoride (PVDF) membrane^22^. Mouse anti-B2L (gifted by professor Keshan Zhang, Lanzhou Veterinary research institute, Lanzhou, China) and mouse anti-kisspeptin antibody (Sigma-Aldrich, USA) in 1:1000 dilutions were used as a primary antibody. Horseradish peroxidase-conjugated (HPR) goat anti-mouse IgG (Abclonal, USA, 1:2000 dilution) was used as a secondary antibody. The reaction was developed in enhanced chemiluminescence reagent in the dark (Pierce, Rockford, IL, USA) and the image was captured using the Image Quant LAS 4000 (GE, Boston, USA).

### Experimental animals

Specific pathogen-free Sprague–Dawley male rats, aged six weeks, with an average body weight of 200 ± 27 grams were purchased from Liaoning Changsheng Biotechnology Co., Ltd and housed in the Center of Laboratory Animals, Huazhong Agricultural University, China. The experimental rats were kept in a pathogen-free room under stable conditions (12-h/12-h dark/light cycle, 50–60% humidity, and ~22 °C temperature). Rats had access to sterilized food and water ad libitum and were acclimated for two weeks before use.

### Ethics statement

The study was carried out in accordance with the guidelines for the care and use of experimental animals for the scientific purpose set by the Ministry of Science and Technology (Beijing, China: No.398, 2006). The institutional animal care and use ethics committee of Huazhong Agricultural University approved the protocol.

### Experimental design and immunization protocol

The experiment was performed in the completely randomized design, and 36 rats were equally assigned into one of the following three groups: PBK-asd, PK-asd and PVAX-asd (control). Both the treatments and the control group rats were treated with (100 μg/dose) plasmid diluted in 1 ml saline. The injection was given through intramuscular route. All treatment rats were boosted twice at an interval of 3 weeks.

### Sample collection

Blood samples were collected from the lateral tail vein at fortnight interval from day 0 of primary immunization to the end of the experiment (12 weeks). The serum samples were collected by centrifuging at 3000 rpm at 4 °C for 10 minutes, and stored at −20 °C. At 12 weeks after primary immunization, rats were euthanized by cervical dislocation and their spleens and testes were aseptically isolated for cell proliferation, flow cytometry, cytokine assay, and testis histology.

### Specific anti-B2L antibody response

Indirect ELISA was used to determine serum anti-B2L antibody according to references^8,24^ with some modification. Briefly, costars plates were coated with 600 ng/well of B2L protein diluted in 100 μl 0.1 M NaHCO_3_ (PH 9.6) overnight at 4 °C. The fluid was aspirated, washed four times with PBS/0.1% Tween-20, and blocked for 90 min at 37 °C with PBS/0.1% Tween-20/2% BSA (200 μl/well). Then, the plates were washed four times, and 100 μl serum diluted at 1:50 in blocking solution was added to each well and incubated at 37 °C. After 1 hour incubation, the plates were washed and 100 μl/well HRP- goat anti-rat IgG (Bioss, Beijing, China) diluted at 1:5000 in blocking solution was added and incubated for 1 h at 37 °C. The reaction was developed by adding 100 μl per well (A + B) TMB substrate solution and reacted at 37 °C for 10 min. Finally, the enzymatic reaction terminated by adding 50 μl/well of 2 M H_2_SO_4_ and the plates were read at OD 450 nm. Results were expressed as an antibody endpoint titer, determined when the OD value is 3-fold higher than the background value obtained with a 1:50 dilution of serum from control rats^8^.

### Lymphocyte proliferation assay

The single-cell suspension was prepared by smashing the spleens using microscopic slides and filtered through a 70 μm nylon membrane^8^. For proliferation assay, the cell density was adjusted to 2 × 10^4^ cells/ml in RPMI 1640 medium supplemented with 10% FBS. Then 100 μl of cell suspensions per well were seeded into 96-well plates. The cells were cultured in triplicate with and without 5 μg/ml B2L protein and 5 μg/ml concanavalin A (Sigma-Aldrich). The cells were incubated for 48 hours at 37 °C followed by adding 10 μl cell count kit-8 (Beyotime, Shanghai, China) into each well and further incubated at 37 °C for 4 h. The absorbance was then measured at 450 nm using an EnSpire multimode plate reader (Waltham, USA).

### Immunophenotyping of CD4^+^ and CD8^+^ T cells

To detect T cell subsets of CD4^+^ and CD8^+^, the cells were adjusted to a density of 1 × 10^7^ cells/ml. Then, 100 ul cells from each sample were centrifuged at 1500 rpm for 5 min and suspended with PBS. After centrifugation at 2000 rpm for 2 min at 4 °C, the cells were washed twice with PBS, containing 0.5% bovine serum albumin (BSA). The cells were stained at 4 °C for 30 min with APC-anti-rat CD3 (Invitrogen), FITC-anti-rat-CD4, and PE-anti-rat-CD8A (Biolegend, San Diego, CA, USA). The stained cells were suspended in 0.5 ml of PBS containing 0.5% BSA and analyzed by flow cytometry.

### Cytokines assay

To determine the levels of orf specific cytokines of tumor necrosis factor-alpha (TNF-α-), interferon-gamma (IFN-ϒ) and interleukin (IL) 2, 4 and 6 produced by T lymphocytes, cell culture supernatants from B2L (5 μg/ml) antigen-stimulated cell were collected and measured using commercial ELISA kits (Dakewei, Beijing, China) according to the manufacturer’s instructions.

### Detection of anti-kisspeptin antibody

The serum anti-kisspeptin antibodies were determined using an indirect enzyme-linked immunosorbent assay (ELISA) with kisspeptin peptide as a coating antigen as previously described^22^. The HPR-goat anti-rat antibody IgG (Bioss, Beijing, China) in 1:5000 dilutions was used as a secondary antibody. The antibody endpoint titers were determined using the formula N > *μ* + 2 SD as previously described^25^. Where: N is the absorbance of the highest serum dilution; *μ* and SD are the mean and standard deviation of negative control samples at the same dilution.

### Hormone measurement

Serum LH and testosterone levels were detected using commercial rat specific ELISA kits (Cusabio Biotech Co., Ltd, Wuhan, China). The specific operation was performed following the kit instruction. The sensitivity of the assay for LH and testosterone was 0.15 mlU/ml and 0.06 ng/ml, respectively. The intra and inter-assay coefficient of variation was <15%.

### Histological analysis of testes

Both testes from each rat were collected; weigh and, their length and width were measured using vernier calipers. One testis from each rat was fixed with 10% neutral buffered formalin (NBF) for histology analysis and stained with hematoxylin and eosin as previously described^26^.

### Data analysis

Data were analyzed using SAS Version 9.3 (SAS Institute, Cary, NC, USA). The anti-kisspeptin antibody, anti-B2L antibody, LH, and testosterone levels were analyzed in the following general linear model using treatment group, sampling weeks, and their interaction as a fixed effect.

$${Y}_{ij}=\mu +{T}_{i}+{W}_{j}+{(TW)}_{ij}+{e}_{ij}$$

Where: *Y*_*ij*_ = observation on anti-B2l antibody, anti-kisspeptin antibody, LH and testosterone levels

*μ* = overall mean

*T*_*i*_ = fixed effect of i^th^ treatment group (PBK-asd, PK-asd, and control)

*W*_*j*_ = fixed effect of j^th^ sampling weeks (week 0, 2, 4, 6, 8 10 and 12)

*TW*_*ij*_ = interaction between treatment and sampling weeks

*e*_*ij*_ = random error

The effect of treatment on testis size and cell proliferation were analyzed using the following GLM procedure:

$${Y}_{i}=\mu +{T}_{i}+{e}_{ij}$$

Where: Yi = observation on testis size and cell proliferation; (μ = overall mean; Ti = fixed effect of i^th^ treatment group (PBK-asd, PK-asd and control) and *e*_*ij*_ = random error.

Multiple comparisons among groups were performed using Tukey test.

The cytokine data were analyzed using student *t*-test. Flow cytometry data were analyzed using flowjo software. The data were presented in mean ± standard error form, and statistically significant was considered when *p* < 0.05.

## Results

### Fusion protein epitope prediction

The 3D structure of the B2L and kisspeptin-54 recombinant gene was predicted using the online https://zhanglab.ccmb.med.umich.edu/I-TASSER/ server and PyMOL software. The 3D structure result showed that the fusion protein is separated and antigenic determinant of B2L and kisspeptin-54 is exposed to the surface that could be accessible to the paratope (Fig. S2).

### Identification of plasmids and fusion protein detection

The recombinant plasmid was identified by restriction digestion using *NheI* and *EcoRI* enzymes (Fig. S3). We confirmed that B2L and kisspeptin-54 gene attached by (G_4_S)_3_ linker was successfully cloned into PVAX-asd vector without an antibiotic resistance gene.

**Figure 1:**
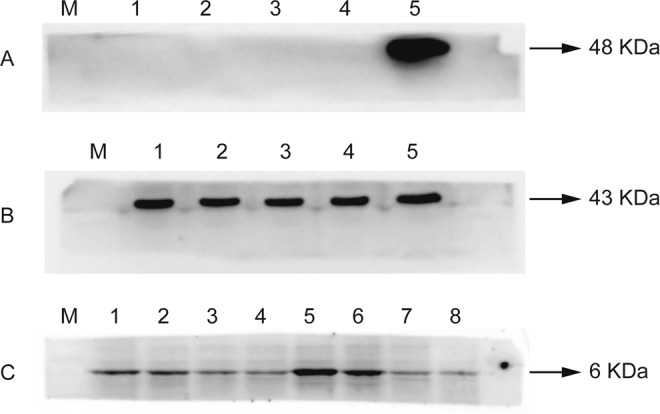
(**A**) The protein expression product of B2L-Kisspeptin-54 fusion protein. Lane M: protein marker; Lane 1 and 2: cell product after transfection with PVAX-asd (negative control); lane 3 and 4: Hela cells (negative control); Lane 5: cell product after transfection with PBK-asd. The band at about 48 KDa at lane 5 is the B2L-kisspeptin-54 fusion protein, no bands were found in the negative controls (Lanes 1 to 4). (**B**) Western blot result of β actin control gene: lane M: protein marker. The bands at about 43 kDa from lane 1 to 5 are β actin. (**C**) The protein expression products of kisspeptin-54. The bands from lane 1 to 8 at about 6 KDa are Kisspeptin-54.

To confirm the expression of the recombinant plasmid, protein was extracted from transfected cultured Hela cells and analyzed using western blot. We detected a protein band at the expected B2L-kisspeptin-54 recombinant protein size (48 kDa) (Fig. 1A) and kisspeptin-54 (6 kDa) (Fig. 1C), indicating that the plasmids could successfully be translated into protein in the mammalian cell.

### B2L specific antibody response

**Figure 2:**
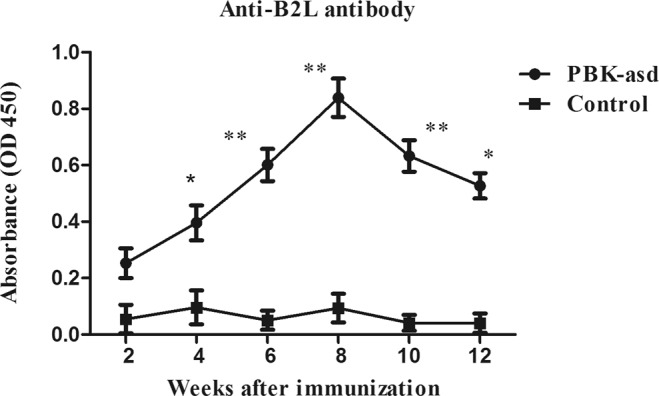
The serum anti-B2L antibody levels in PBK-asd treated and control group rats from 2 weeks to 12 weeks after primary immunization. The asterisk indicates a significant difference between treatment groups within a week at *p* < 0.05.

To evaluate the immunogenicity of the recombinant B2L and kisspeptin-54 DNA vaccine against the orf virus envelop protein (B2L), we measured serum anti-B2L levels using indirect ELISA. The ANOVA results in Table S1 showed significant differences between the PBK-asd immunized and the control group (*p* < 0.01). The ELISA results presented in Fig. 2 showed that the anti-B2L antibody was significantly higher in the PBK-asd group than in the control group from the 4^th^ week after the initial immunization until the end of the experiment (*p* < 0.05). This data suggesting that immunization of rats using recombinant B2L and kisspeptin-54 DNA vaccine could stimulate antibody production against orf virus envelope protein (B2L).

### Cellular immune responses

#### Cell proliferation assay

**Figure 3:**
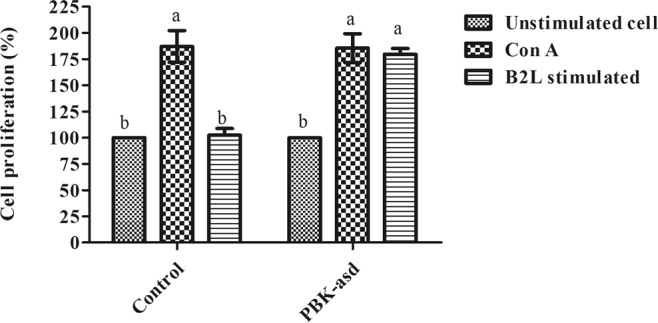
Cell proliferation of splenocytes measured by CCK-8 kit. On average 2 × 10^4^ splenocyte cells were taken from PBK-asd immunize and control group rats. The cells were cultured in triplicate with and with out B2L protein. Then the cell proliferation was measured by adding CCK-8 kit. The mitogen, Concavalin A was used as a positive control. Different superscript letter within the same group indicates a significant difference at *p* < 0.05.

Lymphocyte proliferation is a vital characteristic of the response of lymphocytes to antigenic stimulation. Herein, to examine whether the PBK-asd immunization up-regulates antigen mediated lymphocyte proliferation or not, we measured cell proliferation using CCK-8 assay from spleen cells stimulated with B2L protein. Interestingly, immunization with the recombinant PBK-asd vaccine significantly stimulated B2L-mediated splenocyte cell proliferation in the immunized group as compared to the control group (*p* < 0.05) (Fig. 3; Table S2).

#### Flow cytometry assay

**Figure 4:**
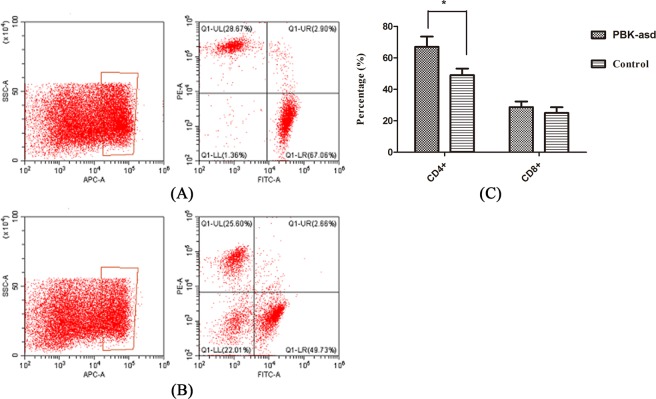
Fluorescence analysis of splenocytes for CD4 and/or CD8 expression from PBK-asd (**A**) and control group (**B**) rats. The spleen cell suspensions from 5 rats of each group were incubated with FITC-labeled rat CD4 mAb and PE-labeled rat anti-CD8 mAb and analyzed by FACSort. The numbers display in the graph indicate the percentages of splenocytes within a section. (**C**) The percentage of CD4^+^ and CD8^+^ lymphocytes in the spleens of the experimental rats. The asterisk indicates a significant difference at p < 0.05.

Cell-mediated immune response aids the host to defense against virus and other pathogens via its help in antibody production and cytotoxic activity. To examine whether the recombinant PBK-asd immunization stimulates a cell-mediated immune response, we measured the T cell subsets of CD4^+^, and CD8^+^ using flow cytometry, and results are displayed in Fig. 4. The data revealed that the PBK-asd recombinant vaccine-induced cellular immunity evidenced by higher CD4^+^ levels in PBK-asd immunized group than those in the control group (*p* < 0.05). However, a significant difference in the CD8^+^ content was not detected between PBK- asd immunized and control group (*p* > 0.05).

#### Antigen-specific cytokine production

**Figure 5:**
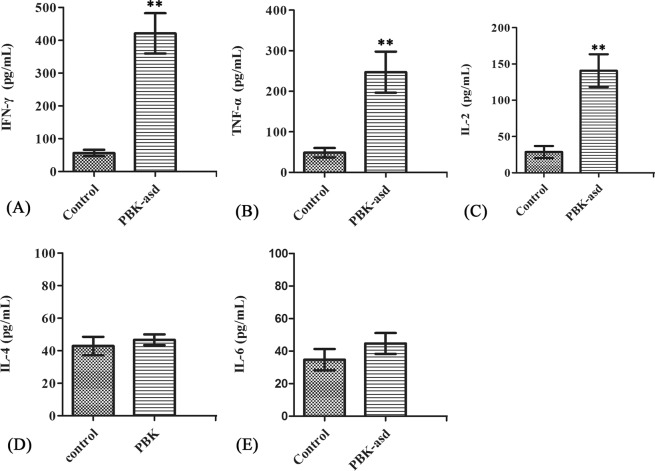
The cytokine production (**A**) IFN-γ, (**B**) TNF-α, (**C**) IL-2, (**D**) IL-4 and (**E**) IL-6 by splenocytes from PBK-asd immunized and control group rats after 48 hours stimulation with B2L protein. The result was determined using ELISA. The *indicates a significant difference at *p* < 0.05. The error bar represents the mean ± S.E.M of cytokines levels.

To further identify the type of CD4^+^ helper cells (Th1 or Th2) produced in response to the vaccine, we detected TNF-α, INF-γ, IL-2, IL-4 and IL-6 cytokines marker from B2L protein stimulated splenocytes. The data revealed that IL-2, INF-γ, TNF-α cytokines were significantly higher in PBK-asd immunized group than those in the control group (*p* < 0.05) (Fig. 5A–C). However, the IL-4 and IL-6 levels in the splenocyte supernatant was not significantly different compared to the PBK-asd treated and control group (Fig. 5D,E) suggested that PBK-asd immunization activated Th1 helper cell but not Th2 helper cell.

### Anti-kisspeptin antibody response

**Figure 6:**
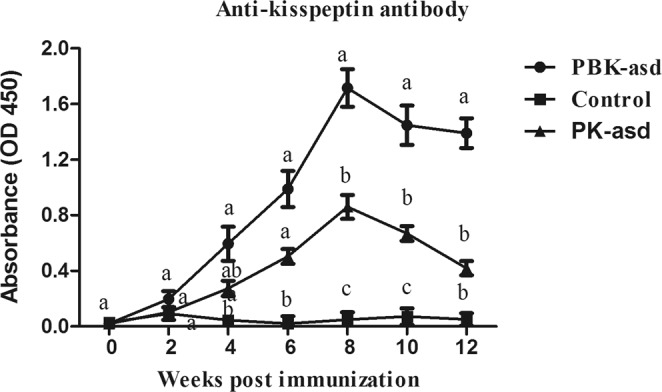
The serum anti-kisspeptin antibody levels in rats treated with PBK-asd (n = 12), PK-asd (kisspeptin-54 alone) (n = 12) and control (n = 12) from primary immunization to 12 weeks post-primary immunization. Different superscript letter among the experimental groups within a week indicates a significant difference at *p* < 0.05

In the aim to determine the antibody response of experimental rats against kisspeptin-54, serum collected from experimental rats was subjected to ELISA analysis. The ANOVA result indicated that treatment group; sampling week and their interaction had a significant effect on serum anti-kisspeptin antibody response (Table S3). The result presented in Fig. 6 demonstrated that the anti-kisspeptin antibody response in PK-asd group showed a significant difference only 6 to 10 weeks after primary immunization as compared to the control group. However, compared with the control group, recombinant PBK-asd immunization significantly increased serum anti-kisspeptin production from the 4^th^ week after the initial immunization to the end of the experiment (*p* < 0.05), indicating that B2L protein is a promised adjuvant to facilitate the immunogenicity of kisspeptin-54.

### Effect on reproductive hormones

**Figure 7:**
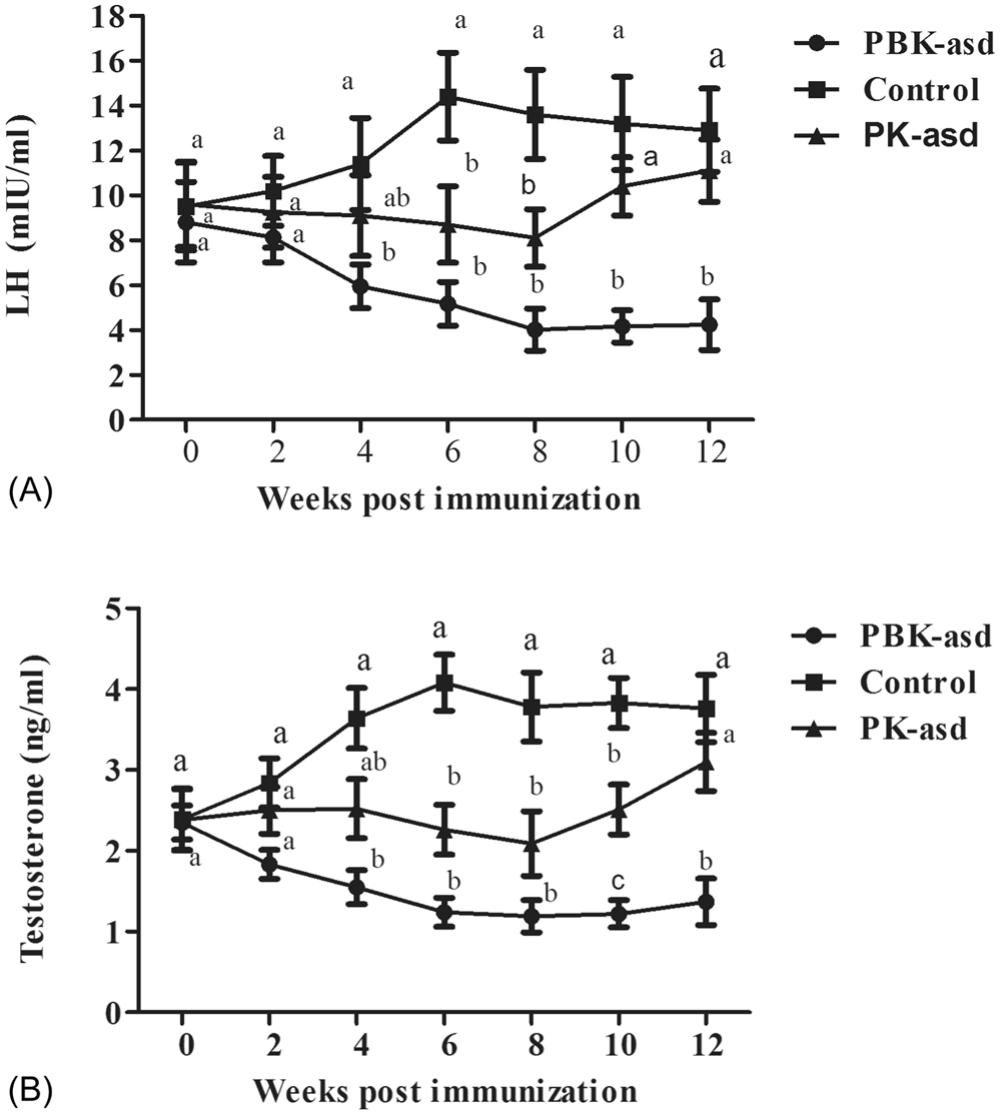
The mean ± S.E.M serum luteinizing hormone (**A**) and testosterone (**B**) levels in rats immunized with PBK-asd (n = 12), PK-asd (n = 12) and control group (n = 12). Different superscript letter among the experimental groups within a week indicates a significant difference at *p* < 0.05.

Luteinizing hormone (LH) and testosterone are essential for sexual development and reproduction in the male; hence, they are good indicators of fertility. In this study, the serum LH and testosterone levels of the experimental rats were determined using ELISA, and results are presented in Fig. 7A,B, respectively. The ANOVA results of LH (Table S4) and testosterone (Table S5) indicated that treatment group, sampling week and their interaction had a significant influence on serum LH and testosterone hormone levels (*p* < 0.01). In PK-asd immunized rats, both LH and testosterone levels decreased and showed a significant difference compared to the control group at week 6 and 8 (*p* < 0.05), but after 8 weeks the LH and testosterone levels reversed and did not show a significant difference (*p* > 0.05). Interestingly, PBK-asd immunization significantly reduced serum LH and testosterone levels compared with the control group, from week 4 to the end of the experiment (*p* < 0.05), suggesting that using B2L as an adjuvant for kisspeptin-54 could achieve long term LH and testosterone inhibition.

### Testes size and morphology

We measured the weight, length and width of the testes to assess the effect of recombinant B2L and kisspeptin-54 vaccine on testicular size. The ANOVA result presented in Table S6 showed that there was a significant difference between the immunized and control group (*p* < 0.05). As shown in Table 1, the PBK-asd vaccine caused testicular atrophy, as reflected by remarkably lower testis weight, length and width in the immunized group. However, a significant difference in testis size was not observed between PK-asd and the control group at 12 weeks after primary immunization.

**Table 1:** The mean ± SEM testicular weight, length and width of experimental rats at 12 weeks after primary immunization.

Parameters	PBK-asd immunized	PK-asd	Control
Testis weight (g)	0.98 ± 0.005^b^	1.67 ± 0.11^a^	1.77 ± 0.14^a^
Testis length (cm)	1.13 ± 0.055^b^	1.83 ± 0.14^a^	1.88 ± 0. 13^a^
Testis width (cm)	0.67 ± 0.058^b^	1.24 ± 0.16^a^	1.14 ± 0.29^a^

Notes: Means with a different superscript small letter in the same row indicates a significant difference (*p* < 0.05).

**Figure 8:**
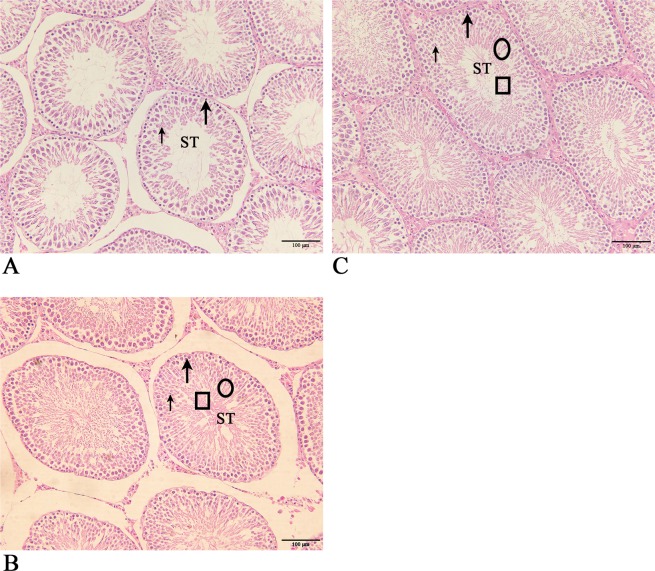
The histological analyses of testes from the PBK-asd immunized (**A**), PK-asd (**B**) and control group (**C**) rats stained with hematoxylin and eosin. The ST represents the seminiferous tubules. The long arrow, short arrow, ellipse, and square indicates the spermatogonia, spermatocytes, spermatid and spermatozoa, respectively. In PBK-asd immunized group, the histology of testes was disrupted as demonstrated by loosely arranged spermatogonia, few spermatocytes and devoid of spermatid and spermatozoa. While, in the PK-asd and control group the spermatogonia, spermatocytes, spermatid and spermatozoa are dense and closely arranged. The magnification power is 20X and the scale bar represents 100 μm.

To examine the effect of the vaccine on testicular histology, testes from experimental rats were stained with hematoxylin and eosin and results are displayed in Fig. 8. The result revealed that spermatogenesis was disturbed by the PBK-asd vaccine evidenced by loosely arranged spermatogonia, few spermatocytes and absence of spermatids and spermatozoa in PBK-asd immunized group (Fig. 8A). In contrast, in the PK-asd (Fig. 8B) and control group (Fig. 8C) dense, tight and closely arranged spermatogonia, spermatocytes, spermatids, and spermatozoa were observed.

## Discussion

Although DNA vaccines are able to induce a strong immune response against a wide range of diseases, concern has been raised about the use of an antibiotic resistance gene as a selection marker during vaccine preparation^27^. In our study, we have successfully constructed a recombinant orf virus B2L gene and kisspeptin-54 DNA vaccine in PVAX I expression plasmid without an antibiotic resistance gene. We have demonstrated that this bivalent DNA vaccine not only provided immunogenicity but also had no risk of an antibiotic resistance gene.

The capability of the vaccine to induce both humoral and cell-mediated immune response defines its efficacy. Our results showed that the recombinant PBK-asd vaccine induced a strong antigen-specific humoral ant-orf antibody after booster immunization.

Splenocyte proliferation is an important aspect of a cell to trigger a cell-mediated immune response^28^. Interestingly, the splenocytes from PBK-asd immunized group was remarkably up-regulated by antigen-mediated stimulation. Our results suggest that the recombinant B2L and kisspeptin-54 combination has the potential to activate T cell immunity.

Cell-mediated immunity response by the infected animals plays an essential role during the recovery period from parapoxvirus^3,29,30^. Herein, the CD4^+^ T cell subset in the PBK-asd immunized group was higher than the control group, implying that the recombinant vaccine could elicit cell-mediated immune response particularly CD4^+^. This result is concurrent with the previous study, which showed that CD4^+^ T cells are the primary cellular effectors that are critical for protection and clearance of orf virus, whereas, CD8^+^ lymphocytes did not appear to be essential for viral clearance^3^.

We measured the secretion of cytokines (IL-2, INF-γ, TNF-α-, IL-4, and IL-6) from splenocytes stimulated with B2L protein. Data showed that PBK-asd immunization significantly raised antigen-specific TNF-α, INF-γ and IL-2 responses in the splenic cell. However, a significant difference in IL-4 and IL-6 levels was not detected between experimental groups. These data demonstrated that PBK-asd recombinant vaccine induced robust cell-mediated immunity, predominantly Th1 helper cells. Our findings are supported by the previous studies reporting that the type 1 (Th1) helper cell particularly IFN-γ cytokine production by lymphocyte is responsible for rapid clearance of the orf virus^7,8,31^. Similarly, recombinant B2L (011) and F1L (059) orf virus gene vaccine had no effect on IL-4 and IL-6 cytokines in mice^8^, indicated that Th2 immune response is not associated with protection against the orf virus^31^. The PBK-asd recombinant vaccine reported here could induce Th1 cell cytokines, which are required for virus clearance.

The recombinant B2L and kisspeptin-54 DNA vaccine produced potent serum anti-kisspeptin antibody. However, in the PK-asd group, the antibody response was low and after 10 weeks, a significant difference with the control group was not observed. Our data demonstrated that a major envelope protein of orf virus (B2L) could be used as an immunomodulator of kisspeptin-54 to produce potent anti-kisspeptin antibody that neutralizes endogenous kisspeptin to inhibit fertility. This result is supported by the previous study in humans showing that low serum kisspeptin levels may be the cause of infertility^32^. Immunization against kisspeptin-54 might reduce fertility by lowering the serum kisspeptin concentrations and disrupting the normal HPG pathways, as evidenced by lower serum kisspeptin levels^33^ and higher anti-kisspeptin antibody^22,23^ in the KISS1 immunized ram.

To further confirm whether the PBK-asd recombinant vaccine had an effect on downstream LH and testosterone production, we measured the serum LH and testosterone levels. Our data demonstrated that both LH and testosterone levels were lower in the PBK-asd immunized group than the control group, suggesting that the vaccine inhibited LH release from pituitary and testosterone synthesis. Whereas, in PK-asd group, both LH and testosterone levels increased after 10 weeks after primary immunization. This indicated that the B2L gene enhances the strength and longevity of anti-kisspeptin antibody production to inhibit the LH and testosterone hormones in PBK-asd group. Our study is in agreement with previous studies using a GnRH vaccine showing that active immunization against GnRH reduced serum LH and testosterone levels in camel^34^, boar^14^, and rat^15^.

The size of the testis reflects the volume of sperm produced. The recombinant PBK-asd vaccine caused testicular atrophy as evidenced by smaller testis weight, length and width. Testosterone, which is the critical hormone in testis growth and development, was reduced due to the PBK-asd immunization, thereby testis growth inhibited and spermatogenesis arrested. In the histological analysis of testis, PBK-asd immunization reduced spermatogonia, primary spermatocyte, and spermatid and abolished sperm production in the seminiferous tubules. This might be due to the reduced concentrations of LH from the pituitary, which was insufficient to initiate testosterone production. Consequently, the morphology of the testis changed and sperm production was inhibited. Likewise, previous studies in young rams immunized with kisspeptin vaccine showed loosely arranged spermatogonia, primary spermatocytes, spermatids and were devoid of spermatozoa^22,33^. Overall, the PBK-asd recombinant vaccine inhibited male fertility via producing robust anti-kisspeptin antibody, reducing the endogenous kisspeptin, and thereby disrupting the normal hormonal secretion in the HPG axis and results in the prevention of spermatogenesis. It is concluded that the recombinant B2L and Kisspeptin-54 vaccine could be a promising approach for the construction of a bivalent orf virus and immunocastration vaccine. Furthermore, we concluded that the orf virus envelope protein (B2L) could be used as an immunomodulator for kisspeptin-54 to produce a strong antibody response.

## Supplementary information

Supplemetary materials

## Data Availability

All datasets generated for this study are available within the article and its Supplementary files.
