# Supplementary material for: Recombinant B2L and Kisspeptin-54 DNA Vaccine Induces Immunity Against Orf Virus and Inhibits Spermatogenesis In Rats
Source: Sci Rep. 2019 Nov 7;9:16262. doi: 10.1038/s41598-019-52744-y (PMC6838309; doi:10.1038/s41598-019-52744-y)
Supplement: Supplementary file 1 — Supplemetary materials [file 41598_2019_52744_MOESM1_ESM.pdf]

Recombinant B2L and Kisspeptin-54 DNA Vaccine Induces Immunity Against Orf Virus and Inhibits Spermatogenesis In Rats

Teketay Wassie[1, 2], Zeng Fanmei[1], Xunping Jiang[1, 2*], Guiqiong Liu[1, 2], Shishay Girmay[1], Zhang Min[1], Liu Chenhui[1], Dong Dong Bo[1], Sohail Ahmed[1]

Supplementary Materials

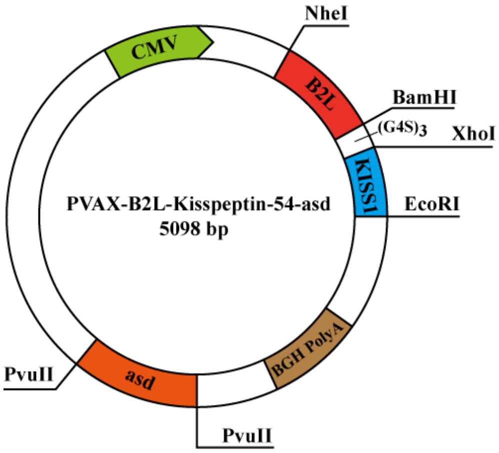

Supplementary Figure. 1. The schematic diagram of the PVAX-B2L-kisspeptin-54-asd plasmid. The kanamycin resistance gene from PVAX1 vector was excised using PvuII double digester enzyme and replaced by ASD gene that interestingly made the constructed plasmid free from antibiotic resistance gene. The orf virus B2L gene and kisspeptin-54 were coupled by $(G_4S)_3$ linker and cloned in PVAX-asd vector between *NheI* and *EcoRI* restriction enzyme sites.

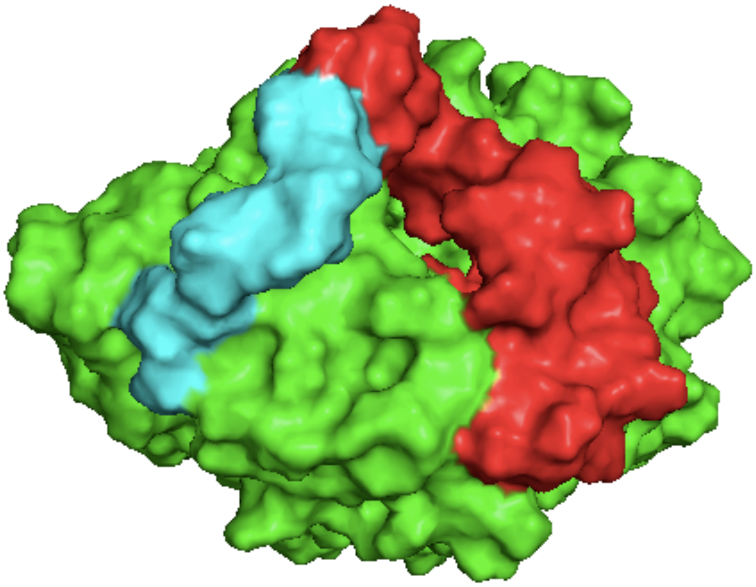

Supplementary Figure 2. The 3D structure of recombinant B2L - (G4S) $_3$-kisspeptin-54.
The 3D structure of the recombinant gene was predicted using the online https://zhanglab.ccmb.med.umich.edu/I-TASSER/ server and PyMOL software. The 3D structure result showed that the fusion protein is separated and antigenic determinant of B2L and kisspeptin-54 is exposed to the surface that could be accessible to the paratope. The green color represents the B2L gene; red color represents kisspeptin-54 and cyan color is the linker (G4S)$_3$.

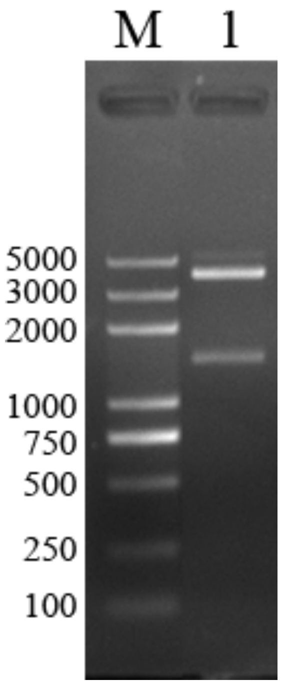

Supplementary Figure. 3. The digested products of the recombinant PBK-asd plasmid. The PBK-asd plasmid was digested by *NheI* and *EcoRI* restriction enzymes. Lane M, the DL5000 DNA marker; lane1: the upper band is PVAX-asd plasmid (3717 bp) and the lower band is the recombinant B2L-$(G4S)_3$ –Kisspeptin-54 (1381bp).

Supplementary table 1. The analysis variance of anti-B2L antibody in the experimental rats.

Source	Type III Sum of Squares	df	Mean Square	F	Sig.
Group	8.258	1	8.258	526.735	.000
Week	1.298	5	.260	16.553	.000
Group * Week	1.198	5	.240	15.281	.000
Error	2.070	132	.016		
Total	26.009	144			

Supplementary table 2. The analysis of variance of cell proliferation in the experimental rats.

Source	Sum of Squares	df	Mean Square	F	Sig.
Between Groups	27874.700	2	13937.350	76.567	.000
Within Groups	2730.436	15	182.029		
Total	30605.136	17			

Supplementary table 3. The analysis of variance of anti-kisspeptin antibody in the experimental rats.

Source	Type III Sum of Squares	df	Mean Square	F	Sig.
Group	28.807	2	14.403	346.735	.000
weeks	21.739	6	3.623	87.222	.000
Group * weeks	16.070	12	1.339	32.238	.000
Error	9.596	231	.042		
Total	126.279	252			

Supplementary table 4**.** The analysis of variance of luteinizing hormone in the experimental rats.

Source	Type III Sum of Squares	df	Mean Square	F	Sig.
Group	7522.812	2	3761.406	253.383	.000
week	490.190	6	81.698	5.504	.000
Group * week	920.968	12	76.747	5.170	.000
Error	2805.662	189	14.845		
Total	46292.031	210			

Supplementary table 5. Analysis of variance result of testosterone hormone in the experimental rats

Source	Type III Sum of Squares	df	Mean Square	F	Sig.
Group	7522.812	2	3761.406	253.383	.000
Week	490.190	6	81.698	5.504	.000
Group * Week	920.968	12	76.747	5.170	.000
Error	2805.662	189	14.845		
Total	46292.031	210			

Supplementary table 6. The analysis of variance result of testis size in the experimental rats.

Source		Sum of Squares	df	Mean Square	F	Sig.
Weight	Between Groups	4.435	2	2.217	280.185	.000
	Within Groups	.261	33	.008		
	Total	4.696	35			
Length	Between Groups	4.186	2	2.093	26.173	.000
	Within Groups	2.639	33	.080		
	Total	6.824	35			
Width	Between Groups	2.225	2	1.113	36.006	.000
	Within Groups	1.020	33	.031		
	Total	3.245	35			
